# Cloning and heterologous expression of cellulose free thermostable xylanase from *Bacillus brevis*

**DOI:** 10.1186/2193-1801-3-20

**Published:** 2014-01-10

**Authors:** Girish K Goswami, Medichtrla Krishnamohan, Vikrant Nain, Chetana Aggarwal, Bandarupalli Ramesh

**Affiliations:** Amity Institute of Biotechnology, Amity University Rajasthan, Jaipur, 302001 India; Department of Biotechnology, Birla Institute of Scientific Research, Jaipur, 302001 India; Department of Biotechnology, Gautam Buddha University, Greater NOIDA, 201308 India; Division of Microbiology, Indian Agricultural Research Institute, New Delhi, 110012 India; Department of Genetics and Plant Breeding, Ch. Charan Singh University, Meerut, 250004 India

## Abstract

Xylanase gene isolated from *Bacillus brevis* was expressed in *E. coli* BL21. Sequencing of the gene (Gen Bank accession number: HQ179986) showed that it belongs to family 11 xylanases. The recombinant xylanase was predominantly secreted to culture medium and showed mesophilic nature (optimum activity at 55°C and pH 7.0). The cell free culture medium exhibited 30 IU/ml xylanse activity. The enzyme did not show any cellulose activity and was active under wide range of temperature (40°C to 80°C) and pH (4 to 9). The enzyme showed considerable thermo stability and regained over 90% of activity, when returned to 55°C after boiling for 5 min. These physiochemical properties of *B. brevis* xylanse show high potential of its applications in paper and pulp industry.

**Electronic supplementary material:**

The online version of this article (doi:10.1186/2193-1801-3-20) contains supplementary material, which is available to authorized users.

## Introduction

Xylan is a major component of hemicellulose of forest and agricultural materials such as hardwood, grain straw, corn cobs and grasses (Dekker and Richards 1976; Wilkie 1979). Xylan can be enzymatically degraded to useful products like xylose (Dekker and Richards 1976; Wang et al. 1980), xylitol, and ethanol (Jeifries 1981; Sung et al., 1993). Xylan is generally insoluble in nature; however, a number of microorganisms with the help of some of their enzymes can readily solubilize xylan. D-xylanase (15 kD to 30 kD) is one of the key enzymes required for the degradation of xylan (Dekker and Richards 1976; Domiano et al. 2003).

One of the exciting prospects for recombinant xylanase is its use in the paper and pulp bio-bleaching (Saleem et al. 2009; Singh et al. 2013) to reduce the requirement of organo-chemicals for bleaching process (Kuhad et al. 1998). However, for its use in paper and pulp industry for bio-bleaching, xylanase pretreatment has to take place at a high temperature and in alkaline conditions; hence thermostable xylanases (Chapla et al. 2012; Saleem et al. 2012) with high pH optimum are of great importance. Moreover, for industrial application xylanase should be celleulase free and require minimum downstream processing for its production. Therefore, this study was carried out to increase the xylanase production by heterologous expression of *Bacillus brevis* xylanase gene in *E. coli* and secreting it in the medium, so that it require minimum downstream processing for its applications in paper and pulp industry. Analysis of some of its biochemical characteristics was carried out.

## Materials and methods

### Bacterial strains and culture media

*Bacillus brevis* strains, obtained from *Bacillus* genetic stock center (BGSC Accession number ATCC8246T) was used in the present study. For xylanase production by *B. brevis* M-9 medium supplemented with 1% xylan was used.

### Cloning of β-1, 4-endo-xylanase gene from *Bacillus brevis*

Total genomic DNA of the bacteria was isolated following the modified procedure of Ausubel et al. (1994) and used for template with PCR primers designed from *Brevibacillus brevis* endo-1, 4-beta-xylanase (xylB) gene (GenBank DQ100303). The PCR primers used were as follows; Forward 5′CGG*GGTACC****TAA***ATGTTTAAGTTTAAAAAGAATTTCTTAGTTGG; Reverse 5′ CG*GAATTC*TTACCACACTGTTACGTTAGAACTTCC. The *Kpn*I and *Eco*RI restriction sites incorporated for cloning are *underlined*. The forward primer has a stop codon (Bold highlighted) just before the start codon. It was incorporated to remove N-terminal S.tag fusion sequence present in the pET29 vector and to create a mini-cistron before the xalanse coding sequence. The PCR was conducted in a GeneAmp PCR System 9700 (PE Biosystems) for 35 cycles (each cycle consisting of 1 min of denaturation at 93°C, 1 min annealing at 55°C, 1 min extension at 72°C, and a final 10 min extension at 72°C) using Taq Polymerase (Fermentas). Amplified PCR products were extracted from agarose gels by QIAGEN gel extraction kit and cloned in pUC vector. The sequence verified gene was further subcloned in protein expression vector pET29A using the *Kpn*I and *Eco*RI sites and mobilized to *E. coli* BL 21(DE3) for protein expression.

### Enzyme assays

The xylanase activity was determined with 1% birchwood xylan in 50 mM phosphate buffer of pH 7 at 55°C using the method described by Bailey et al. (1992). The enzymatic reaction was carried out for 5 min and the reducing sugars were determined using the DNS method (Miller 1959). The xylanase activity was measured in terms of international units (IU). One IU of xylanase is defined as one μ mole of xylose produced by 1 ml undiluted enzyme in 1 min. The μ moles of xylose produced by xylanase were deduced from xylose standard plot.

### SDS-PAGE and zymogram

SDS-PAGE (12.5%) was performed by the standard methods as described by Laemmli (1970). Protein bands visualized by incubating gels with gentle shaking for 30 min in 10% trichloroacetic acid, 4 h in Coomassie brilliant blue R250Coomassie blue (0.025%) in (45:45:10 water:ethanol:acetic acid), and overnight in destaining solution (67% water, 25% ethanol, 8% acetic acid). Modified gels for the detection of *in-situ* xylanase activity (zymograms) were prepared by substituting a boiled solution of birchwood xylan (0.5%) for water during the preparation of the separating layer of the gel. Following electrophoresis, these gels were incubated for 1 h with gentle agitation in 2.5% Triton X-100 and then for 30 min at 80°C in pre-heated buffer (12.5 mM bis-tris propane, pH 6.0, at 80°C). *In- situ* xylanase activity was detected by staining the gels for 30 min in 0.1% Congo red and then destaining them in 1 M NaCl. The activity gels were rinsed in a dilute acid solution (10 mM HCl) to increase the contrast between the hydrolyzed (clear) and non-hydrolyzed (black) xylan prior to photography.

For the zymogram analysis, the crude enzyme samples were electrophoresed as above on SDS-PAGE containing xylan (0.1%). After running, the gel was washed four times for 30 min in 100 mM phosphate buffer (pH 7.0); the first two washes containing 25% isopropyl alcohol, to remove SDS and renature protein in the gel. The gel was then incubated for 20 min at 37°C before soaking in Congo Red solution for 5 min at room temperature and washing with 1 M NaCl until excess dye was removed from the active band. The zymogram was prepared after soaking the gel in 0.5% acetic acid solution. The background turned dark blue, and clear zones were observed in the areas exposed to xylanase activity (Nakamura et al. 1994).

### Homology modeling

*B. brevis* xylanse protein structural model was developed through homology modeling using I-Tasser server (Roy et al. 2010). Quality of predicted structural models were evaluated through stereochemical parameters of Ramachandran Plot and verify-3D (Laskoswki et al. 1993; Luthy et al. 1992).

## Results

### *B. brevis*xylanase gene

**Figure 1:**
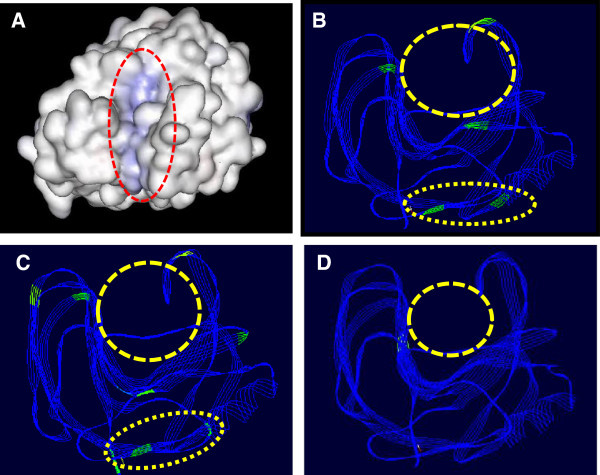
**Structural comparisons of** ***B. brevis*** **xylanase with other** ***Bacillus Sp.*** **(A). Surface view of Xylanase protein 3D structure, (B). Superimposition of two** ***B. brevis*** **structures, Green regions shows differences in the amino acid sequences of the two structures.** Three differences are around active site while two are grouped at the distant region of the proteins. **(C & D)**. Superimposition with B*. licheniformis* and *B. amyloliquefaciens* xylanse 3D structures respectively.

**Figure 2:**
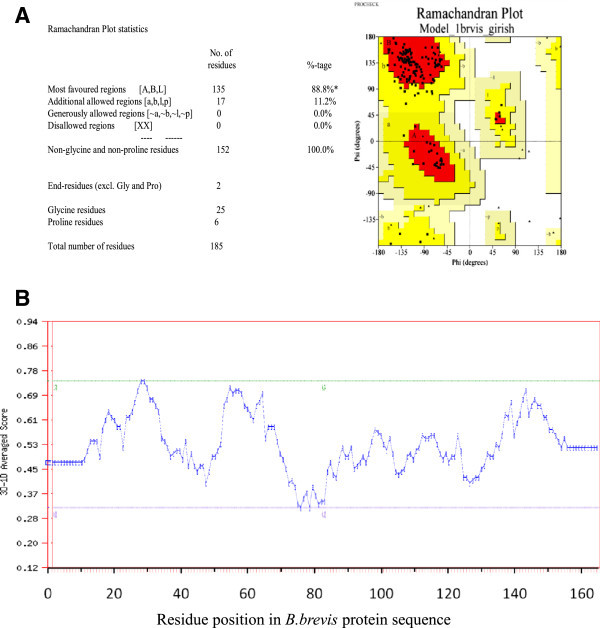
**Stereochemical analysis of predicted of** ***B. brevis xylanase*** **structural model. (A)**. Ramachandran plot analysis of predicted structure model of *B. brevis xylanase*. **(B)**. Evaluation of *B. brevis xylanase* structural model with verify-3D.

The xylanase sequence obtained in the present study was aligned with the published sequence of xylanase from *B. brevis* (GenBank DQ100303)*.* The nucleotide sequence alignment shows that both the sequences align completely without any gap (Additional file 1: Figure S1). However, there is a difference of 33 nucleotides between these two xylanase sequences. Alignments of translated sequences of these two proteins reveal a difference in seven amino acids (Additional file 2: Figure S2). Whether this difference is reflected in 3D model or not was compared by developing the 3D model of both Xylanses followed by superimposion (Figure 1A and B). These two structures differ only marginally from each other. High-lighting of differences on the 3D structure show that most of the differences in amino acid residues are present in two clusters. One around the active site and second below the site (Figure 1B). To understand weather the clustering of difference in amino acid sequences present in 3D model is restricted to *B. brevis* or same pattern is also present in other species, xylanase sequences from different *Bacillus* species were retrieved from the database and their 3D structure developed and evaluated. Results summarized in Figure 1C show that this pattern of clustering of mutation around active site is present in other *Bacillus* xylanase also (Figure 1C and D). The *B. Brevis* Xylanase protein sequence was further analyzed for presence of secretary signal peptide. The sequence analysis shows that a 28 amino acid long secretary signal peptide is present in the *B. brevis* Xylanse (Additional file 3: Figure S3). *In silico* sequence analysis and stereochemical evaluation of modeled *B. brevis* xylanase protein 3D structure shows high quality of predicted structural model, without any residue in disallowed region (Figure 2).

### Heterologous expression of *B. brevis*xylanase

**Figure 3:**
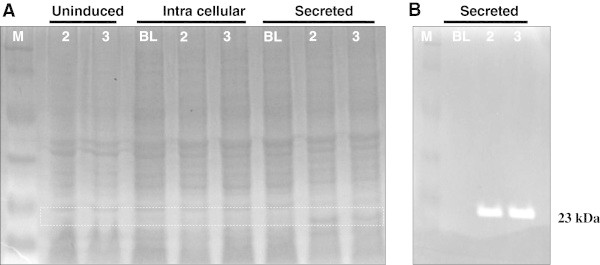
**SDS PAGE analysis for xylanase. (A)**. SDS-PAGE analysis of *B. brevis* xylanase expression in two clones (2&3). M: Protein ladder BL: BL21 (DE3) host cell transformed with pET29A vector (Control), 2&3 are two different colonies for pET-29-xylanse transformed in BL21 (DE3). Xylanse protein in dominantly visible only in medium, indicating that most of protein is getting secreted in the growth medium. **(B)**. Xymogram analysis confirms tthat the 23 kDa protein band observed in SDS is a functional Xylanase enzyme.

**Figure 4:**
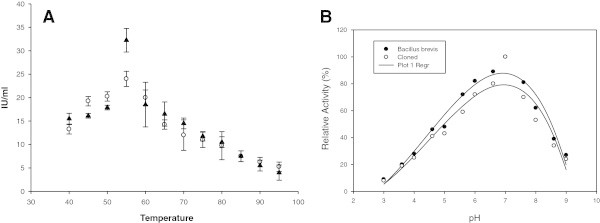
**Effect of temperature and pH on the xylanase activity. (A)**. Effect of temperature on enzyme activity (in IU) of *B. brevis xylanase*. **(B)**. Effect of different pH levels on *B. Brevis* xylanase relative activity.

SDS-PAGE of IPTG induced Zymogram was developed that showed xylanase activity on the gel by clearing zone development on staining with Congo Red dye. Xylanase production was found more (approximately 1.5 times higher) in cloned expression host as compared to *B. brevis* (Figure 3). The optimization of temperature of both cloned and native (*B. brevis*) xylanases show mesophilic nature of *B. brevis* xylanase with maximum activity at 55°C Figure 4A. Similarly the optimum pH of the *B. brevis* xylanase was found at pH 7 (Figure 4B). After finding optimum temperature and pH, effect of high temperature on the enzyme activity was measured. Although enzyme showed considerable loss of enzyme activity at higher temperature and negligible activity was observed above 90°C, the enzyme regained more than 90% activity after boiling for 5 min and subsequent cooling at 37°C for 45 minutes. The enzyme was also evaluated for any cellulose activity and as expected, no detectable cellulase activity was observed.

## Discussion

Many xylanase genes have been isolated from different microbial organisms and were expressed in *E. coli*, however, the xylanase expression level in *E. coli* was generally lower than the parent organism (Bernier et al. 1983; Gallardo et al. 2003; Paice et al. 1986; Panbangered et al. 1983, 1984, 1985; Sandhu and Kennedy 1984; Sipat et al. 1987) and confined mainly to the cytoplasmic or periplasmic fractions (Kulkarni et al. 1999). Hyperexpression of a *B. circulans* xylanase gene in *E. coli* was reported by (Yang et al. 1989) with a xylanase activity of 7 IU/ml found in intracellular fraction of *E. coli*. Extra-cellular activity of xylanase had also been reported earlier in recombinant *E. coli* for the bacterial xylanases from alkaliphilic *Bacillus* by Honda (1985), and alkaliphilic, and thermophilic *Bacillus* species by Shendye and Rao (1993). A synthetic gene encoding mature *B. circulans* xylanase has been designed to imitate the frequency of degenerate codons used in *E. coli*. This synthetic gene was then converted to *B. subtilis* xylanase gene via substitution of Thr^147^ Ser codon. Under the control of *lac* promoter, recombinant xylanase activity in solution form in cytoplasm was reported as high as 300 mg/ml (Sung et al. 1993).

In the present study we have isolated a xylanase gene from *B. brevis* and cloned it in *E. coli*. We understand that this is the first report on cloning and heterologous expression of *B. brevis* xylanase gene in BL21 host. The most significant part of this study is the expression of cloned xylanase gene in extracellular medium of *E. coli* which may be exploited for production of xylanase at commercial level. It is interesting that the xylanase activity in the *E. coli* clone is higher (2 times) over that of native *B. brevis* strain, possibly due to higher level of protein expression. The enzyme produced by *E. coli* is functionally active and capable of degrading birchwood xylan even in SDS-PAGE gel also. *In silico* sequence analysis and stereochemical evaluation of modeled *B. brevis* xylanase protein 3D structure shows high quality of predicted structural model, without any residue in disallowed region (Figure 2). Further comparison of xylanses from different bacterial sources reveals high level of structural similarity with point mutations. These point mutations might have evolved to provide adaptations to bacteria under different environmental conditions. Further studies on the DNA sequence of the cloned fragment containing xylanase gene should prove helpful in determining the regulatory sequences of the gene and the subunit structure of this enzyme.

## Conclusion

*B. brevis* xylanase was efficiently expressed and secreted by *E. coli* (BL21). The xylanase activity was found more (2 times) in culture filtrate of BL21 as compared to *B. brevis*. Heterologous expression systems that produce large amounts of secreted proteins with in an organism that can be grown in industrial scale fermenters must be developed to facilitate higher enzyme production by using agri-waste as a carbon source for enzyme production. Since optimization of growth medium is a useful tool to attain high levels of enzyme activity at lower cost (Damaso et al. 2003) further studies on medium optimization might improve the yield of *B. brevis* xylanase production with the kind of BL21 expression systems described in this paper. An important further consideration is to develop higher-level enzyme producing integrant *E. coli* strains with a number of copies of *B. brevis* xylanase for growth in optimized mefdia.

## Electronic supplementary material

Additional file 1: Figure S1: Nucleotide sequence alignment of published *B. brevis* (*B. brevis*_o) and new *B. brevis* (*B. brevis*_G) xylanase sequences. identical sequences are highlighted in boxes. (PPT 111 KB)

Additional file 2: Figure S2: Amino acid sequence alignment of published *B. brevis* (*B. brevis*_O) with the new *B. brevis* (*B. brevis*_G). Identical residues are highlighted in the box. (PPT 86 KB)

Additional file 3: Figure S3: Identification of signal peptide in *Bacillus brevis* xylanase. (PPT 158 KB)
