# Supplementary figures and images for: Cloning and heterologous expression of cellulose free thermostable xylanase from Bacillus brevis
Source: Springerplus. 2014 Jan 10;3:20. doi: 10.1186/2193-1801-3-20 (PMC4320173; doi:10.1186/2193-1801-3-20)

## Slide 1
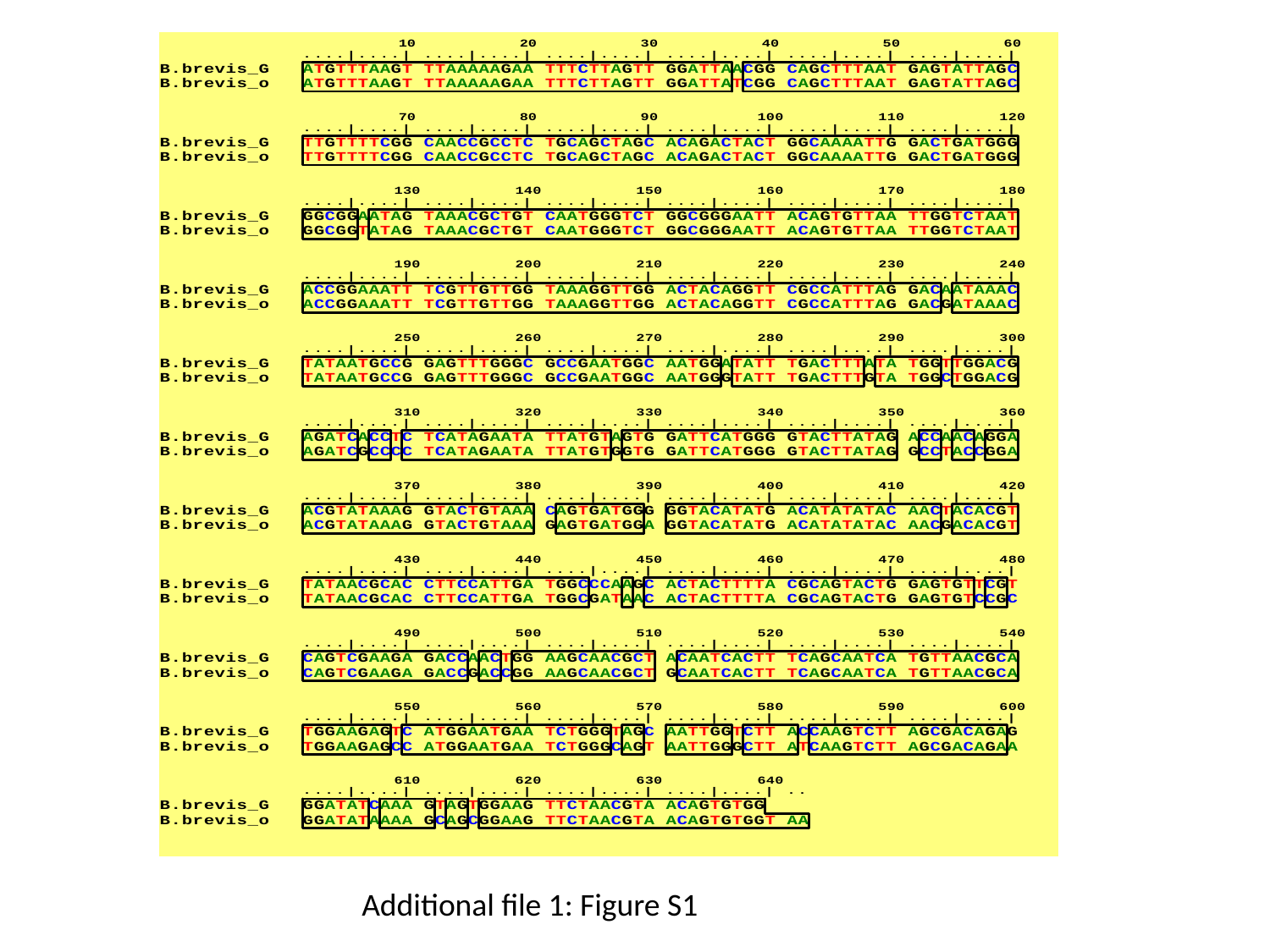

Additional file 1: Figure S1

Supplement: Supplementary file 1 — Additional file 1: Figure S1: Nucleotide sequence alignment of published B. brevis (B. brevis_o) and new B. brevis (B. brevis_G) xylanase sequences. identical sequences are highlighted in boxes. (PPT 111 KB) [file 40064_2013_1451_MOESM1_ESM.ppt]

## Slide 1
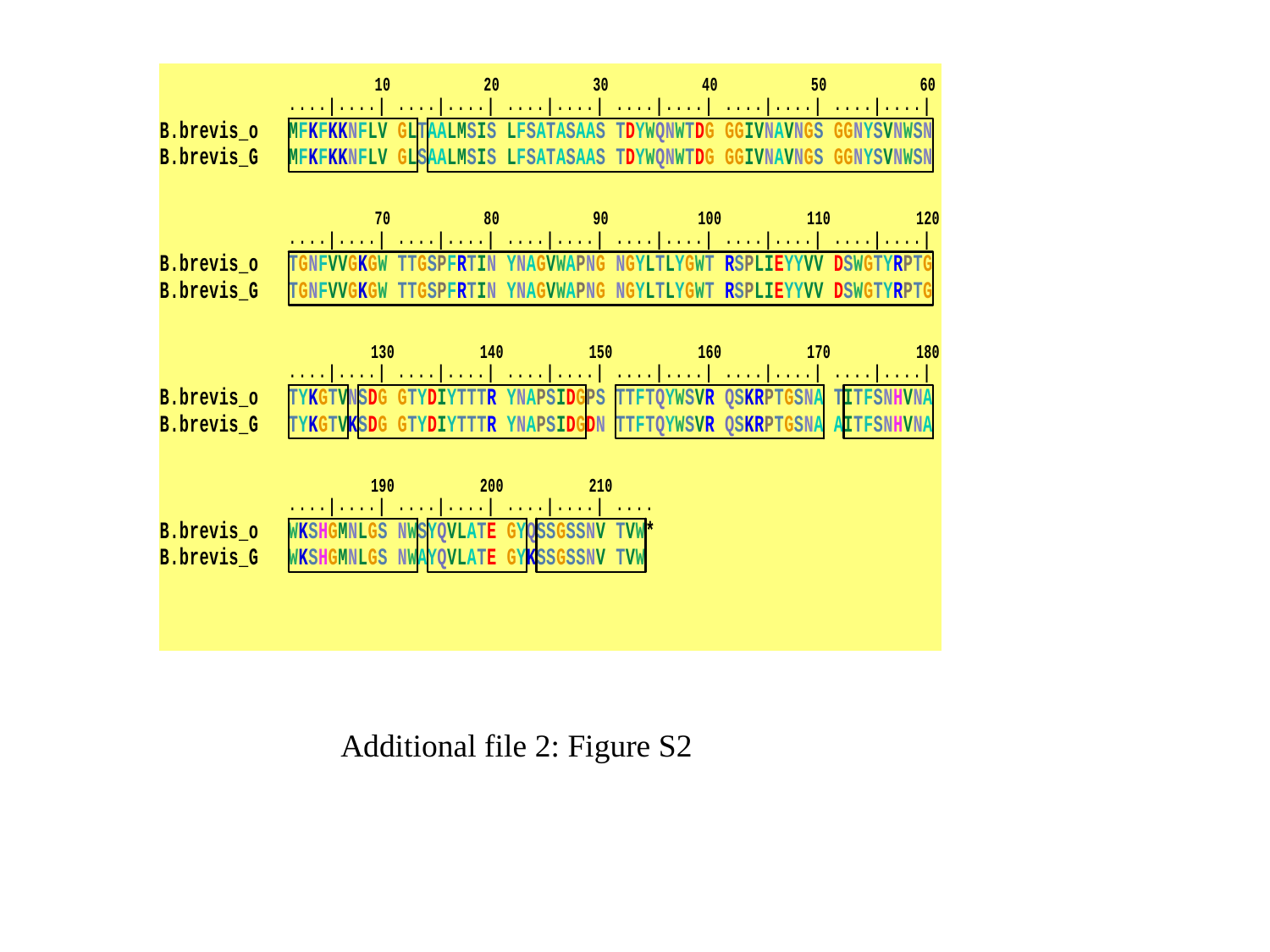

Additional file 2: Figure S2

Supplement: Supplementary file 2 — Additional file 2: Figure S2: Amino acid sequence alignment of published B. brevis (B. brevis_O) with the new B. brevis (B. brevis_G). Identical residues are highlighted in the box. (PPT 86 KB) [file 40064_2013_1451_MOESM2_ESM.ppt]

## Slide 1
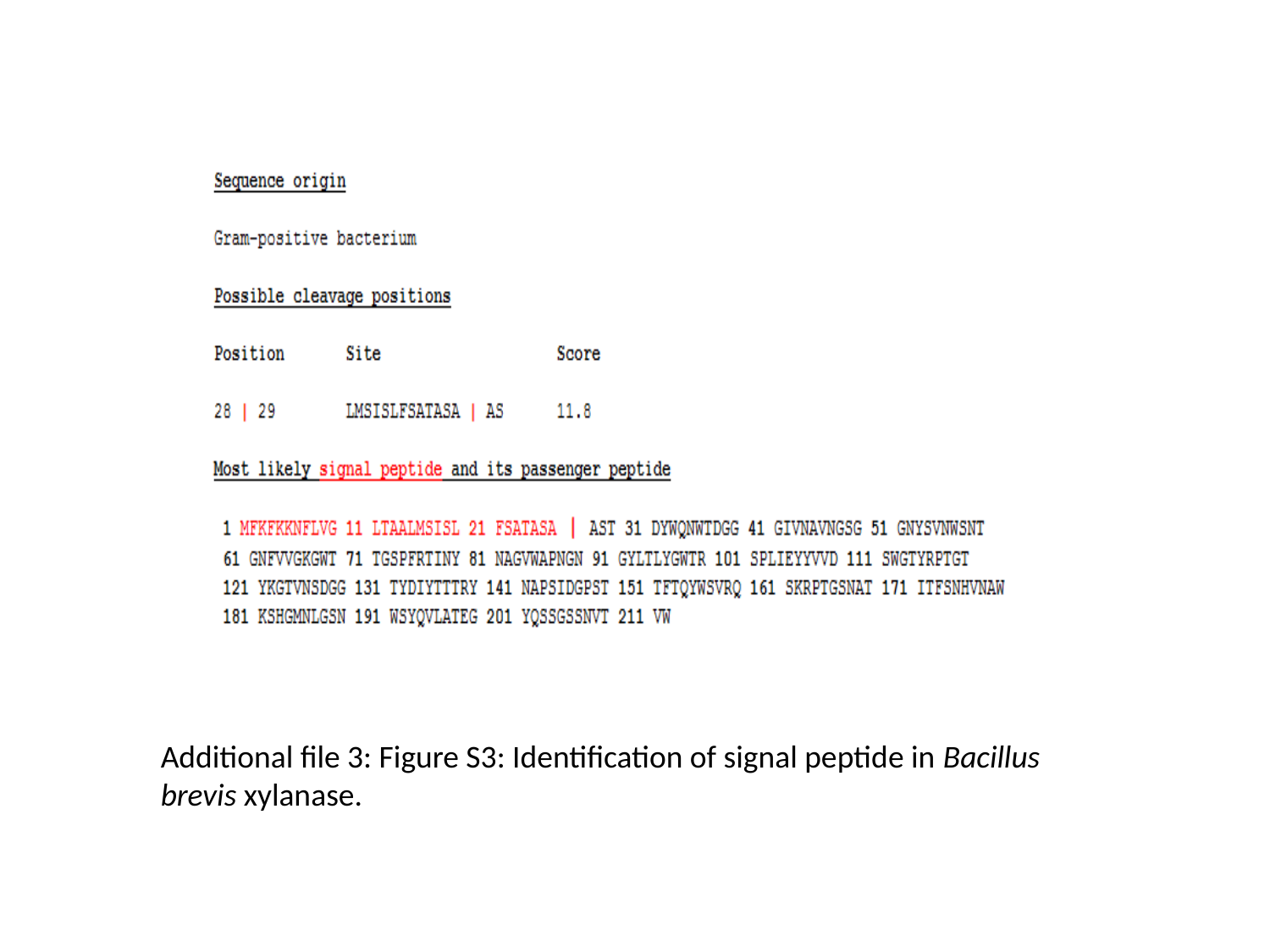

Additional file 3: Figure S3: Identification of signal peptide in Bacillus brevis xylanase.

Supplement: Supplementary file 3 — Additional file 3: Figure S3: Identification of signal peptide in Bacillus brevis xylanase. (PPT 158 KB) [file 40064_2013_1451_MOESM3_ESM.ppt]
